# Comparable antiviral capacity but favorable in vitro survival of CD8s from elite controllers compared to untreated progressors

**DOI:** 10.1186/1742-4690-9-S2-P286

**Published:** 2012-09-13

**Authors:** D Shasha, F Porichis, K Daniel, O Angiuli, DE Kaufmann, B Walker

**Affiliations:** 1 The Ragon Institute of MGH, MIT and Harvard, Boston, MA, USA

## Background

CD8s inhibition of viral replication in vitro was demonstrated as one of the best laboratory correlates to HIV control. However, past studies invariably used CD8s which were rested in vitro for few days before antiviral capacity examined. This might suggest that the difference between CD8s from elite controllers (EC) and chronic progressors (CP) is not in their inhibition capacity but in their ability to retain cytotoxicity during prolonged incubation. Here we compared cytotoxicity and apoptosis of CD8s immediately after their purification (“fresh CD8s”) or 3 days after in vitro rest (“old CD8s”).

## Methods

Samples from 10 EC, 10 CP, 5 HAART treated and 5 HIV negative patients were examined. “Fresh” and “old” CD8s were used as effectors in viral inhibition assays. HIV-specific CD8s were quantified using tetramer staining. Annexin V binding was used to evaluate apoptosis.

## Results

Using “old” CD8s inhibition capacity was higher among EC compared to CP (logP24 reduction 1.225 vs 0.238, p=0.044). For both EC and CP inhibition was much stronger using “fresh” CD8s, but no significant difference was found between EC and CP (logP24 reduction: 3.13 vs 3.85, p=0.29). HIV negative subjects showed no inhibition using “fresh” or “old” CD8s. IL-2 partially rescued antiviral capacity of rested CD8s. Loss of HIV-specific CD8s measured by tetramer staining was higher in CP compared to EC with up to 10-fold increase in Annexin V binding.

## Conclusion

HIV-specific CD8s from CP are endowed with an unexpectedly strong viral inhibition capacity when examined directly ex vivo. CD8s from EC and CP mediated similar HIV suppression directly ex vivo, while the superior antiviral activity of CD8s from EC after a 3d incubation was associated with better survival of HIV-specific CD8s. The capacity to survive and exert effector functions over extended periods, rather than the intrinsic antiviral capacity, best distinguishes CD8s from EC and CP.

